# A critical evaluation of PI3K inhibition in Glioblastoma and Neuroblastoma therapy

**DOI:** 10.1186/2052-8426-2-32

**Published:** 2014-10-27

**Authors:** Mike-Andrew Westhoff, Georg Karpel-Massler, Oliver Brühl, Stefanie Enzenmüller, Katia La Ferla-Brühl, Markus D Siegelin, Lisa Nonnenmacher, Klaus-Michael Debatin

**Affiliations:** Department of Pediatrics and Adolescent Medicine, University Medical Center Ulm, Ulm, Germany; Department of Neurosurgery, University Medical Center Ulm, Ulm, Germany; Department of Pathology and Cell Biology, Columbia University Medical Center, New York, NY USA; Laboratorio Analisi Sicilia Catania, Lentini, SR Italy

**Keywords:** PI3K, Glioblastoma, Neuroblastoma, Cancer, Pharmacological inhibitors, Signaling cascade

## Abstract

Members of the PI3K/Akt/mTor signaling cascade are among the most frequently altered proteins in cancer, yet the therapeutic application of pharmacological inhibitors of this signaling network, either as monotherapy or in combination therapy (CT) has so far not been particularly successful. In this review we will focus on the role of PI3K/Akt/mTOR in two distinct tumors, Glioblastoma multiforme (GBM), an adult brain tumor which frequently exhibits PTEN inactivation, and Neuroblastoma (NB), a childhood malignancy that affects the central nervous system and does not harbor any classic alterations in PI3K/Akt signaling. We will argue that inhibitors of PI3K/Akt signaling can be components for potentially promising new CTs in both tumor entities, but further understanding of the signal cascade’s complexity is essential for successful implementation of these CTs. Importantly, failure to do this might lead to severe adverse effects, such as treatment failure and enhanced therapy resistance.

## Introduction

Modern cancer therapy aims at eradicating tumors by predominately inducing apoptosis, a form of programmed cell suicide, via DNA damage and is thus limited by emerging resistance of cancerous cells and toxic side effects on healthy tissue. In order to circumvent these limitations, combination therapy (CT) that combines cytotoxic agents, such as chemo- or radiotherapy at metronomic doses with at least one molecular-targeted agent, i.e. a pharmacological inhibitor of signaling cascades, has been proposed to break apoptosis resistance, while concurrently lowering adverse side effects [1]. The PI3K/Akt/mTOR survival signaling cascade is frequently considered a promising target in modern CT, particularly as PTEN, its negative regulator, is among the most frequent mutated proteins in cancer [2]. Small molecule compounds that inhibit members of the PI3K signaling network, either pan-specific, on the level of individual protein classes or even isoform-specific, are being developed and clinically evaluated by several companies [3, 4]. However, despite promising preclinical data, clinical successes so far have been limited. There is mounting evidence that the discrepancy between preclinical and clinical data is due to the intrinsic complexity of the PI3K/Akt/mTOR signaling cascade, so that the reductionist view of PI3K signaling as a mere linear 'survival pathway' seems no longer appropriate:

1. Signaling cascades, such as the PI3K/Akt/mTOR pathway, are often depicted as a linear cascade, their activation akin to a row of dominoes falling, with individual signaling arms running parallel (at the same speed) with occasional feedback loops. However, the reality is more in line with a Rube Goldberg Machine. These pathways have evolved out of more primitive cascades, often with different functions, have recruited proteins and whole side arms and thus created overlapping or even diametrically opposite functions. A signaling cascade is not maintained in a population – be it mammal, human or tumor – due to its efficacy or elegance, but remains active in a population if it is the best current option that a) does what it needs to do and b) is available to said population. This, in turn, means that there is no simple linear relationship between degree of inhibition and tumor cell death. In other words our data, published and unpublished, as well as others clearly indicate maximal PI3K inhibition does not equal maximal sensitization for cell death [5, 6].
2. Taking the evolutionary origins of cellular signaling pathways into account, the notion of a survival pathway that can be inhibited by taking out a central component appears greatly simplistic. The notion of distinct signaling cascades that overlap is a human construct, probably not dissimilar to the Linnæan classification system, useful most of the time, but not reflecting the whole biological truth. For example, there is mounting evidence that the PI3K/Akt/mTOR pathway and the Ras/MEK/Erk signaling cascade are – at least in some cellular systems – so entwined that one could really view them as a single, even more complex survival machinery (with a canopy of additional functions) [7]. Therefore, blocking an individual component is not comparable to figuratively blowing up a railroad bridge and thus stopping the cancer train, but more like blocking the highway forcing cancer to take the byroads and local diversions, slowing down progression, but with the minor risk of the discovery of a novel shortcut.

Taking these two caveats into account our answer to the question *Is the PI3K signaling network still a promising target for cancer therapy?* is *Yes, but it is not the magic bullet hoped for*.

## Review

### Glioblastoma and PI3K signaling

Glioblastoma multiforme (GBM) is the most common primary tumor of the central nervous system in adults [8] and with a mean patient survival of 15 months after treatment initiation, it is considered to be among the most lethal cancers [9]. The current standard of care consists of tumor resection followed by radiotherapy and a course of Temozolamide [10]. As PTEN expression is reduced by mutation or loss of heterozygocity in 5-40% and 60-80%, respectively [11], and PI3K/Akt/mTOR signaling is elevated in ~88% of all glioblastoma [12, 13], the addition of pharmacological inhibitors to current treatment schedules should lead to clear therapeutic benefits. Indeed several clinical trials have been initiated to study this hypothesis [14], but most have led to no significant therapeutic improvement, or – in the case of bevacizumab – have yielded rather controversial results [15]. So far, aside from two mTOR (complex 1) inhibitors, Everolimus and Temsirolimus, no drug targeting the PI3K/Akt/mTOR pathway specifically has been approved [14].

The first interesting point of note is that inhibition of PI3K signaling does not lead to apoptosis induction, although this has been reported for other tumor entities, such as Hodgkin lymphoma [16]. Furthermore, as soon as patients are treated with Temozolamide the presence or absence of PTEN within the tumor is irrelevant for any future prognostic development [11], the same phenomenon holds true for activated Akt [17]. In contrast, activity of the mTOR arm of the signaling cascade is associated with poor patient survival [17]. However, due to its complex role in several cellular processes [18], in particular autophagy, which may lead to increased or reduced cell survival [19] and the upregulation of ERK signaling upon its inhibition [20], mTOR is generally considered a difficult target in glioblastoma [21]. Furthermore, frequently the promising *in vitro* data regarding mTOR inhibition in Glioblastoma cells does not translate well into an *in vivo* setting (for example [22]).

Our own initial data analyzing a panel of Glioblastoma cell lines responding to CT combining inhibition of PI3K signaling and various chemotherapeutic agents, as well as death receptor ligands also yielded several interesting points [23, 24]: PI3K signaling contributes to therapy resistance in GBM cell lines, independently of PTEN status. However, this contribution seems stronger when cells are additionally stressed, i.e. serum-starved. Importantly, the observed effects often do not appear particularly strong, making it hard to argue that the PI3K/Akt/mTOR signaling cascade is the main mediator of apoptosis resistance in GBM. The above described clinical data seem to support these inferences, which leads to the question: Why then is this pathway so frequently mutated in GBM? We suggest three possible reasons that might explain this apparent contradiction. They are by no means mutually exclusive and are all supported by the literature.

1. Activation of PI3K signaling functions as a driver mutation for the cell of origin to re-acquire stem cell characteristics/re-enter the cell cycle. While the cell of origin is still strongly debated in GBM [25], primary GBM, the more common form making up 91-95% of all GBM [26], apparently arise *de novo* within 3–6 months [26], suggesting rapid proliferation in a tissue which generally exhibits rather little cell division. This is of particular interest if GBM does not arise from neural stem cells or oligodendrocyte precursor cells [27], but de-differentiated astrocytes [25], and would fit with the observation that inhibition of PI3K signaling GBM primary affects proliferation [28, 29]. The most compelling data for this hypothesis comes from work in T cell acute lymphoblastic leukemia where it could be shown that activated AKT signaling enhanced the frequency of leukemia propagating cells, which can be considered in this context as tumor stem cells [30].
2. PI3K/Akt facilitate the invasive phenotype which is characteristic for GBM, both in terms of motility and survival under stress. An association with FAK and Src has long been established for PI3K (for example [31, 32], thus linking it to signaling complexes associated with adhesion, motility and invasion [33]. Recent data seem to link Akt/mTOR activity directly to GBM motility [34], which is of particular interest as this would suggest a connection between two almost ubiquitous features of GBM, high activity of the PI3K signaling cascade and tumor dissemination throughout the whole brain [35, 36].
3. The central role of PI3K/Akt/mTOR in GBM biology has led us grossly to underestimate its importance. As shown above PI3K has clearly other roles besides survival in GBM, it contributes to motility (point 2) and proliferation (point 1). This would suggest that PI3K signaling has several contradictory functions in GBM. For example, high proliferation is usually associated with therapy sensitivity [37], yet inhibition of PI3K signaling can lead to both reduced proliferation [28, 29] and chemosensitization [23, 24]. This notion does not negate the use of pharmacological PI3K inhibitors in CT, but rather indicates the design of CT needs to be carefully considered.

Interestingly, the importance of the last point is emphasized by our work mainly conducted on a different tumor system, Neuroblastoma (NB).

### Neuroblastoma and PI3K signaling

NB is a common childhood neoplasia of the sympathetic nervous system that is generally characterized as a highly heterogeneous disease and categorized into four stages, of which stage 1 and 2 have a good prognosis. However, prolonged survival for patients with stage 3 and 4 is only 18-30% [38, 39]. Unfortunately 45% of patients exhibit high-risk tumors, most of which have already metastasized at clinical presentation [40]. Interestingly, NB lacks the mutations in PI3K signaling that characterize GBM and other tumors and a role for PI3K in NB was first considered due to its close association with the mycN oncogene [41] and its prominent role downstream of growth factor-initiated signaling, such as IGF-1 and IGF-2 [42]. More recently, the role of the ALK receptor tyrosine kinase has also been highlighted in NB. It is expressed in over 90% of all NB [43] and found to be activated by mutation in 6.9% [44]. One of the downstream survival cascades activated by ALK is PI3K/Akt signaling [42]. Our own group previously showed that in NB phosphorylated Akt correlates with poor patients' prognosis [45], while others subsequently demonstrated a link between PI3K signaling and growth/survival [46], as well as resistance to chemotherapy [47].

However, more recently following some previous hints concerning the underlying molecular mechanisms of how PI3K signaling affects chemosensitivity in NB [48], we were surprised to discover that the relationship between PI3K signaling and chemosensitivity is not as simple as we had assumed [5]. Maximal, prolonged inhibition of PI3K signaling did not sensitize NB cell lines for Doxorubicin-induced apoptosis. Indeed under certain conditions even a desensitization could be observed [5]. This might be due to a combination of the potent anti-proliferative effect of PI3K/mTOR inhibition in NB cells and the induction of autophagy [5], although it should be pointed out that autophagy in NB is controversially discussed, as in most tumor entities, and can be a survival mechanism as well as a cell death enhancer (for example [49, 50]). While concurrent treatment with a chemotherapeutic agent and a pharmacological inhibitor of PI3K signaling led mostly to good apoptosis sensitization, it was the application of the inhibitor several hours after the cell death inducer that yielded the best results [5]. We traced this particular effect to the mitochondria, i.e. the modulation of VDAC1 via Akt-mediated phosphorylation of GSK3β [5].

Importantly, as we found a similar temporal effect in GBM cells, this led us to propose a model which de-emphasizes the maximal inhibition of a target signaling cascade (clinically: high plasma levels of a pharmacological inhibitor prior to chemotherapy) and concentrates on the temporal relationship of the two treatment components, i.e. sequential dosing [5].

### Pathway complexity: when and where?

Next, one needs to consider at which point in the signaling cascade to intervene. While in the past a preferred target seems to have been the dual inhibition of PI3K and mTOR [3], thus taking out a major section of the signaling cascade and ablating a potent feedback mechanism, currently there seems to be a shift towards inhibitors of Akt [4]. However, as emerging data suggest that Akt is not the central mediator of PI3K, i.e. inhibition of Akt signaling is not identical with inhibition of PI3K signaling [7], this target appears less promising. In particular, inhibition of PI3K can also block Ras/ERK signaling, while inhibition of Akt does not and is thus less efficient [7].

To consider the optimal target of PI3K signaling inhibition one has first to understand its role. Our data [5] provides strong evidence for the third proposition we put forward, indicating that we have previously underestimated the complexity of PI3K signaling. Importantly, this does not necessarily invalidate the other two options. In a model we and others [5, 6] suggest the PI3K signaling cascade regulates two or three aspects of cellular behavior relevant for the success of CT: On the one hand proliferation and metabolism (the inhibition of which can have adverse effects on chemo- and radiotherapy [5, 6], e.g. hyperactivation of Akt in this context has even been proposed as a therapeutic option [51]), on the other hand survival, particularly under stress. Importantly, these competing effects of the PI3K signaling network are mediated by different arms of the cascade at different speeds, giving us two therapeutic strategies to employ.

The approach we – and also, apparently, the Djuzenova group – favor is the use of existing, clinically evaluated pharmacological inhibitors of the PI3K signaling, while optimizing the treatment schedule so as concurrently to maximize the effect on survival and minimize the effect on proliferation/metabolism [5, 6]. Alternatively, a strategy could be envisioned whereby not the main components of the PI3K signaling cascade are targeted, but either proteins that are further upstream, apical to several, potentially compensatory networks, such as the MEK/Erk signaling cascade [7], or further downstream at the level of molecules that clearly only contribute to survival in the context of CT, such as the Bcl-2-family [48].

**Figure 1:**
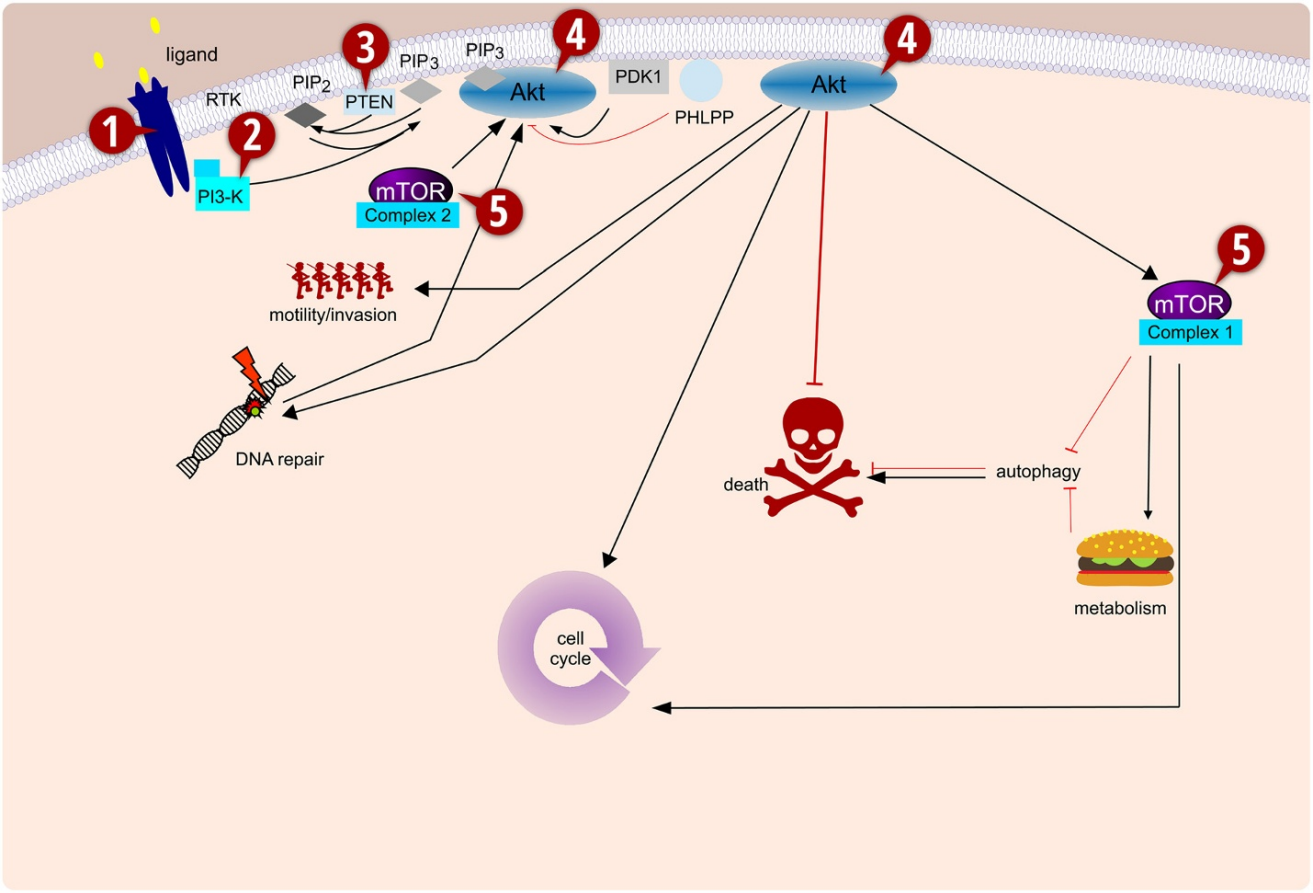
**The PI3K/Akt/mTOR signaling cascade.** Simplified schemata of the PI3K/Akt/mTOR signaling cascade. While indisputable involved at several levels, directly as well as indirectly with cell survival, other important functions of this signaling network are also highlighted here. Key molecules which have been implicated in cancer or have been selected as potential therapeutic targets are: **1.** Receptor tyrosine kinases, such as EGFR, IGFR and ALK, often overexpressed or activated by mutations in many different cancers, including glioblastoma and neuroblastoma. These transmembrane proteins are apical of several interconnected signaling cascades. **2.** PI3K, phosphatidylinositol 3'-OH kinase, a lipid kinase, predominately consisting of a p110 catalytic and a p85 regulatory subunit, of which the former has been found to be mutationally activated in certain cancers. **3.** PTEN, phosphatidylinositol (3,4,5)-triphosphate [PtdIns(3,4,5)*P*_*3*_] phosphatase and tensin homologue, is a phosphatase that counters PI3K-mediated phosphorylation and as such functions as a negative regulator of the PI3K/Akt/mTOR signaling cascade. It is among the most frequently inactivated, either by mutation or promoter methylation, proteins in cancer. **4.** The serine/threonine kinase Akt, v-akt murine thymoma viral oncogene homologue (Protein Kinase B), is often considered the central downstream mediator of PI3K signaling, as it phosphorylates a diverse array of targets that are involved in all key functions of this pathway. **5.** mTOR, mammalian target of Rapamycin, depending on its complex partners, can either be up- or downstream of Akt. It is frequently found to be associated with the process of autophagy, which depending on the context, can be either a survival strategy or a form of cell death. (modified from [33]).

The epidermal growth factor receptor (HER1/EGFR) is one of the most frequently dysregulated oncogenes in glioblastoma [52]. About half of GBM show amplification of HER1/EGFR and of these about another half coexpress EGFRvIII, the continuously activated mutant form of the receptor [53]. In preclinical studies, treatment with HER1/EGFR-targeted agents such as monoclonal antibodies or small-molecule inhibitors, e.g. erlotinib (Tarceva; Genentech Inc.) were shown to exert promising antineoplastic activity in various *in vitro* and *in vivo* models in the setting of GBM [52, 54, 55]. These effects were at least in part related to inhibition of PI3K/Akt and MAPK signaling. However, when taken into the clinics HER1/EGFR inhibitors did not hold up to the expectations derived from the promising preclinical results [56–58]. Treatment with erlotinib did not result in a survival benefit either as a single agent therapy [58] or when combined with temozolomide and radiation [57]. Thus, at this point, upstream targeting of PI3K by inhibition of HER1/EGFR did not fulfill hopes in improving the fate of patients with GBM. One potential explanation for this finding is that PI3K signaling may be uncoupled from upstream control by inactivation of PTEN, the previously mentioned negative regulator of PI3K signaling (Figure 1).

*In vitro*, Fan and coworkers showed that PTEN mutant U87 cells were much less responsive to a treatment with erlotinib alone when compared to PTEN wild type LN229 cells [59]. Others reported similar findings [60]. In both studies, additional inhibition of either mTOR by rapamycin or PI3K/mTOR by PI-103 resulted in a sensitizing effect on PTEN mutated glioma cells towards HER1/EGFR inhibition by erlotinib. However, in a clinical setting, the results of a phase I/II pilot study of 22 patients with recurrent GBM treated with the mTOR inhibitor everolimus and the HER1/EGFR inhibitor gefitinib were rather mixed [61].

While identification of potential upstream targets seems less advanced in NB than in GBM, ALK presents itself as a promising candidate, but – although pharmacological inhibitors such as crizotinib are available – preclinical and early clinical trials do not appear promising. It seems that an almost complete inhibition of ALK is needed for a clinical response, while resistance to the inhibitor can develop relative rapidly, as is also the case with HER1/EGFR [62]. Similar to the combination of an HER1/EGFR inhibitor with blocking mTOR signaling in GBM, the combination of mTOR and ALK inhibition in NB is also of therapeutic benefit [63].

These findings in GBM and NB clearly indicate that CT with several inhibitors (of a single signaling network) of which some are upstream of PI3K can lead to enhanced therapeutic responses.

**Figure 2:**
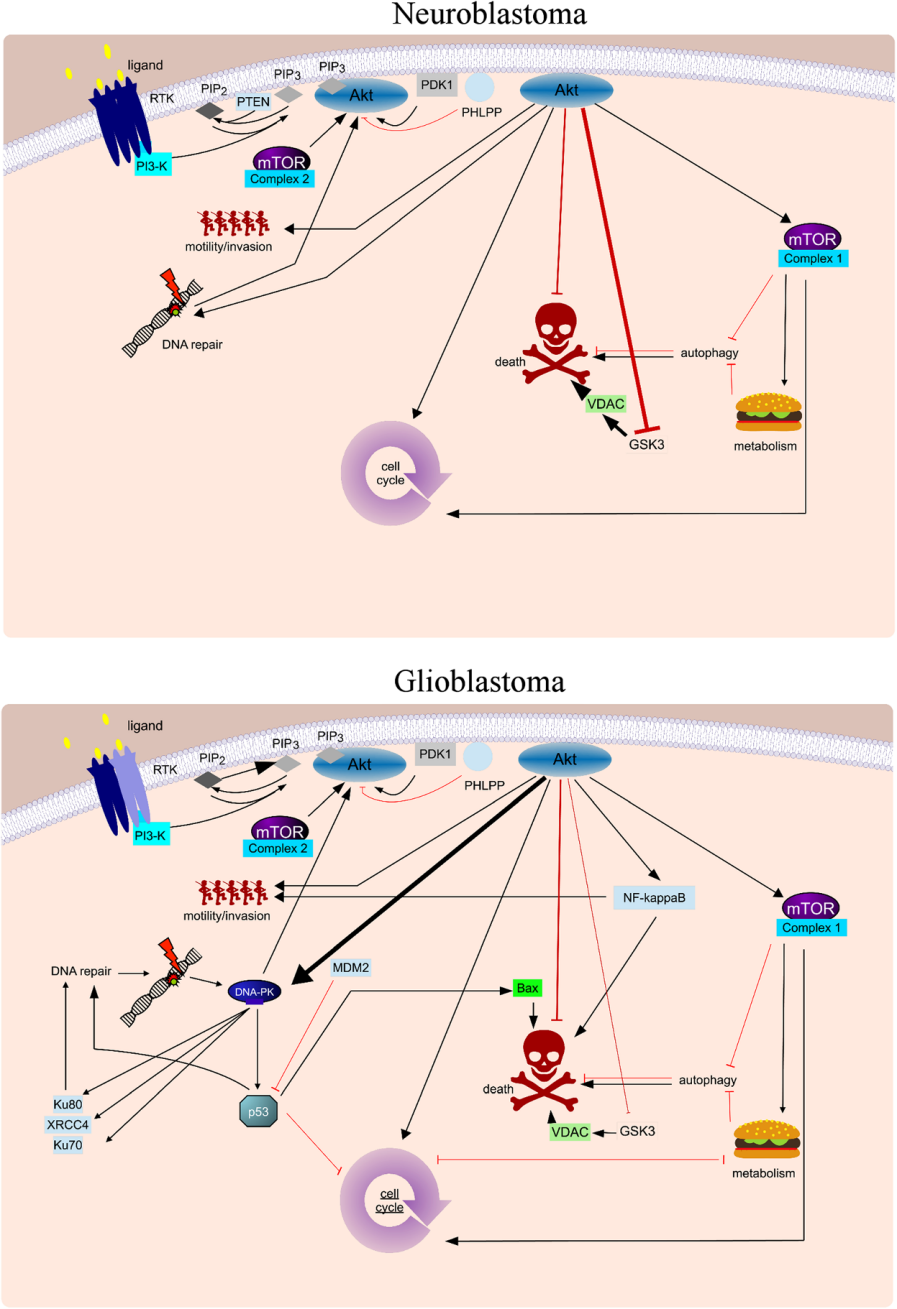
**Potential tumor-specific differences in therapeutic end points.** Although inhibition of PI3K/Akt/mTOR signaling is a feasible approach in both glioblastoma and neuroblastoma, the molecular background of those two malignancies is very different. While glioblastoma exhibits amplification of HER1/EGFR with coexpression of the activated EGFRvIII variant [53], their main feature with regards to the PI3K signaling network is the frequent inactivation of PTEN [11]. In contrast, neuroblastoma does not exhibit any frequent mutation within the PI3K/Akt/mTOR signaling cascade, but often displays overexpression of several receptor tyrosine kinases [42, 43]. Interestingly, our own data suggest that the downstream effectors that need to be inhibited to chemosensitize these tumors to chemotherapy are different. Glioblastoma is sensitized for doxorubicin-induced apoptosis via blocking DNA-PK activation, i.e. DNA repair [24], while in neuroblastoma this seems to be mediated at the level of mitochondria via VDAC1 [5]. It remains to be seen whether this is an isolated phenomenon, due to low numbers of cell lines investigated, or whether different arms of the PI3K network have a different weighting dependent on the malignancy.

To avoid the inhibition of additional side arms of the signaling cascades or activation of compensatory mechanisms rather specific individual targets should be considered, but therefore the precise executor molecule for survival needs to be identified. We have identified DNA-PK, i.e. the DNA repair axis of PI3K signaling, as the crucial inhibitor of survival when GBM cells are challenged with Doxorubicin [24]. However when NB are treated with the same drug GSK-3 and VDAC, i.e. the mitochondrial arm of PI3K signaling, are crucial [5] (Figure 2). Further experiments are needed to find out whether these differences are indeed due to the specific tumor entity, the interplay between treatment and genetic make-up, or different role of the PI3K signaling cascade in different cells. As long as these questions are unanswered downstream molecules, despite the presumed lower side effects, remain undesirable, particularly, as a single end-point target increases the risk of mutational escape, as seen, for example, with Gleevec [64].

**Figure 3:**
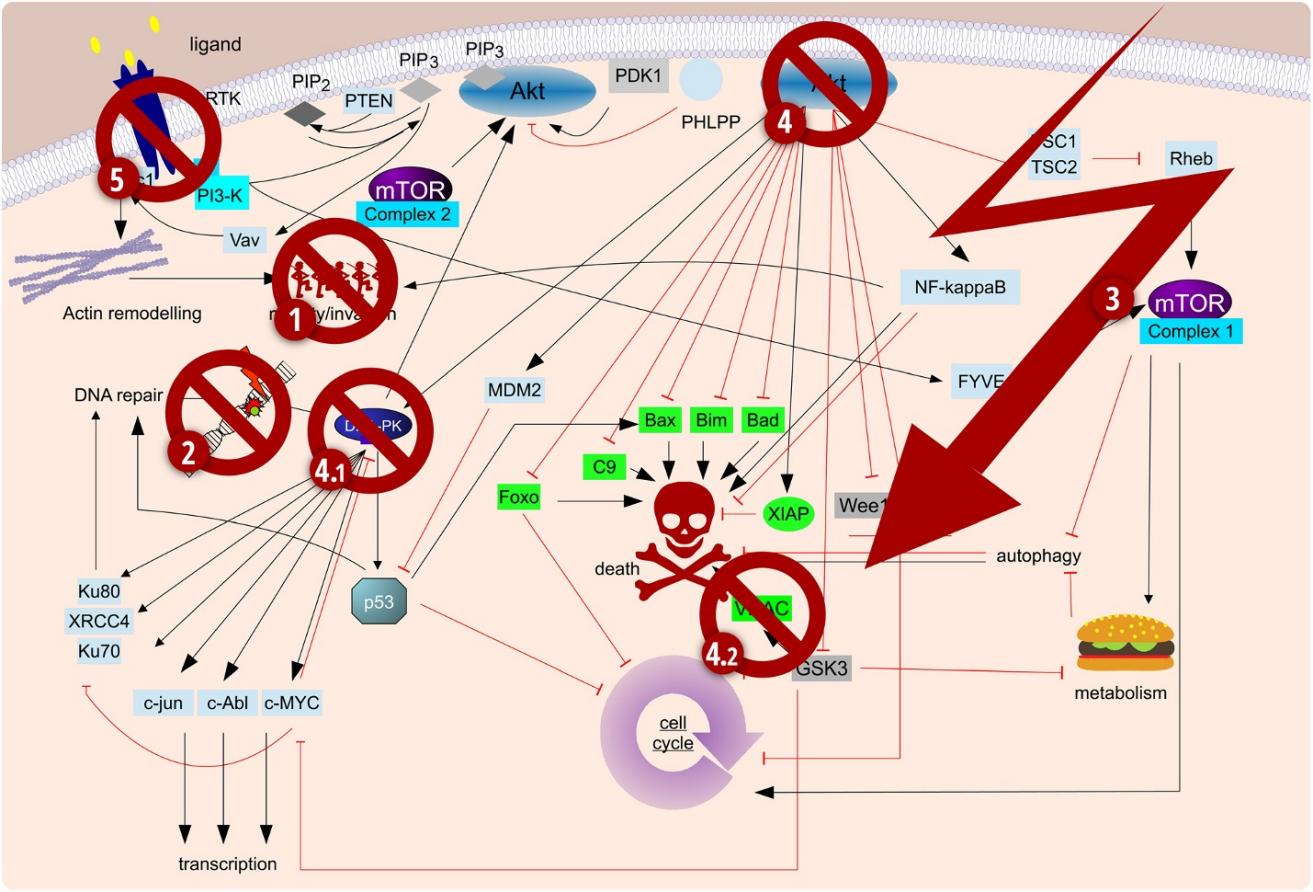
**Possible future treatment schedule.** A possible complex combination therapy that consists of a cell death inducer and several sensitizer that all target individual components of the PI3K signaling cascade. **1.** Take out the cancer cells' ability to move, thus preventing invasion and metastasis, extending the therapeutic window. Continue blocking this arm throughout therapy. **2.** Block the DNA repair mechanisms prior to treatment. Importantly do not affect cell cycle progression, thus making cancer cells amenable to most standard treatments. **3.** Administer chemo- or radiotherapy. **4.** Block the survival pathways mediated by PI3K signaling in the cancer cells stressed by treatment. If, as outlined in Figure 2, details of key mediators are know, e.g. DNA-PK in glioblastoma (4.1), or VDAC1 in neuroblastoma (4.2), target these, otherwise Akt seems the most promising target. **5.** Finally, block growth factor receptors to maximize cell cycle arrest, thus preventing a cancer repopulation by the cells that escaped treatment-induced apoptosis. Point 5 might appear superfluous, as 3 and 4 should already block proliferation, however, this inhibition should also target cells that have activated additional signaling cascades or have (epi)genetically escaped sensitivity to treatment.

Importantly, our data suggest that one does not have to pick a single or – in case of the dual PI3K/mTOR inhibitors - a double target, but that multiple, carefully timed inhibitions of what is generally considered a single signaling cascade can cooperate and thus produce a greater sensitizing effect [5]. Therefore it is feasible, from a purely biological perspective, to imagine a therapeutic approach that uses a stepwise inhibition of PI3K signaling both to extent the therapeutic window and enhance cell death (Figure 3). How such an approach could be translated into a clinical setting and whether cumulative inhibition of signaling might lead to unforeseen side effects are the key remaining challenges that need to be overcome.

## Conclusions

Crosstalk between proneoplastic signaling pathways (or, depending on the perspective: underestimated complexity of a single signaling network) is abundant in both GBM [65] and NB [66, 67] and is regarded as one of the major culprits responsible for the failure of targeted therapies. In addition, it has also become apparent in the last decade or so that the number of mutations found in cancers is considerably higher than estimated: While only a handful are needed to initiate tumor progression, an excess of 10,000 mutations is found in many malignancies [68]. Although many of these will not contribute to the malignant phenotype and might even be present in benign neoplasms [68], other mutations will affect the cancer cells' behavior. With such a complexity, it is not surprising that blocking a single signaling cascade, either alone or as part of a CT approach, has not been successful. Indeed, viewed in this context it is actually surprising what a potent tool inhibition of PI3K signaling is!

As a consequence, multi-targeting has been developed as a therapeutic strategy to overcome this potential mechanism of resistance by combining two or more agents targeting different oncogenic signaling pathways, or targeting a single oncogenic signaling pathway differently in the sense of a combined forces alliance driving the cancer cells to undergo cell death [5, 69–72]. Frequently, the inhibition of PI3K signaling is considered a promising contributor to CTs.

In single therapy inhibition of PI3K signaling often leads to a cytostatic effect, not only in the aforementioned Hodgkin lymphoma [16], but also in both GBM [28, 29] and NB [41, 73]. We have argued elsewhere [33] that an approach that aims to chronify a malignancy rather than an attempt to cure a patient can be the preferable strategy, while others have also pointed out that prolonged cytostasis can ultimately lead to a cytotoxic event, if the tumor cells fail to escape this proliferation block [74]. For CT an antiproliferative effect of the sensitizer is often considered a hindrance, as it has often been suggested that rapid proliferation coincides with increased treatment sensitivity [37]. This is by no means an universal truth [75]. In addition, there are several inducers of cell death which are of therapeutic interest that exert their effects in a proliferation-independent manner, such as death receptor triggering [76, 77], or treatment with amiloride derivatives [78]. Most crucial, as outlined above, there is an emerging line of argument that the carefully timed application of sensitizer and inducer in CT can drastically affect the outcome [5, 6], suggesting that a carefully timed sequential application of inhibitor and sensitizer, together with carefully selected targets for inhibition, quite possibly several targets may lie within the same cascade, can greatly enhance the anti-tumorigenic effect of CT. In such a setting, pharmacological inhibition of PI3K/Akt/mTOR may prove to be an invariable tool in our therapeutic arsenal.

While this review focuses on glioblastoma and neuroblastoma, the inhibition of PI3K-mediated signaling is currently also being clinically evaluated in several additional tumor entities, such as lymphocytic leukemias, colorectal cancer, head and neck cancer, several forms of lymphoma, non-small cell lung cancer and renal cancer [79]. Indeed, it is well-worth remembering that PTEN, the negative regulator of this signaling cascade has often been cited as the second most frequently mutated gene in cancer and even as 'a new guardian of the genome' [80]. Since neuroblastoma and glioblastoma are, with respect to PI3K/Akt/mTOR signaling, two very different entities, there is no *a priori* reason to assume that other tumors will not be subject to the principles discussed here. For example, sequential dosing, has already been demonstrated to also be important in other malignancies, such as breast cancer [81]. However, further research will be needed to fully clarify this point.

With the current rapid advancement of sequencing techniques [82, 83], profiling [84, 85] and molecular histology [8], our understanding of which targets in which signaling cascade might represent promising targets will become much clearer within the next few years. However, other challenges remain, such as how successfully to translate the preclinical data into a clinical setting. For example, our own cell culture work suggested that in a complex CT approach to treat childhood GBM the inhibition of PI3K was preferable to targeting mTOR, however, applying these two combinations to an orthotopic xenotransplant mouse model indicated that the CT that blocked PI3K had no beneficial effect on mouse survival, while the therapy that targeted mTOR significantly prolonged survival [72]. Importantly, on analysing the tumours, we found indications that suggested treatment failure of the first combination was due to an additional effect on tumour vasculature that prevented the chemotherapeutic agent from reaching the malignancy [72]. This effect would have been undetectable in both cell culture and under local instead of systemic treatment of the mice. In addition, while a cell culture model and – to a certain extent – a preclinical animal model enable the precisely sequential-timed application of clearly defined concentrations allowing us fully to understand which amount of a substance reaches the target within what time frame, this luxury is not given in the clinic. A systemic application of treatment is here the norm and thus the precise understanding and control of the complex pharmacokinetics will be essential.
